# Editorial: Plant disease management in the post-genomic era: from functional genomics to genome editing, Volume II

**DOI:** 10.3389/fmicb.2023.1203870

**Published:** 2023-05-10

**Authors:** Sabrina Sarrocco, Alfredo Herrera-Estrella, David B. Collinge

**Affiliations:** ^1^Department of Agriculture, Food and Environment, University of Pisa, Pisa, Italy; ^2^National Laboratory of Genomics for Biodiversity, Center for Research and Advanced Studies of the National Polytechnic Institute, Irapuato, Mexico; ^3^Section for Microbial Ecology and Biotechnology, Department of Plant and Environmental Sciences and Copenhagen Plant Science Centre, University of Copenhagen, Copenhagen, Denmark

**Keywords:** plant disease management, functional genomics, genome editing, metagenomics, pathogen-targeting RNAs

Following the success of the first edition of the Research Topic “*Plant Disease Management in the Post-Genomic Era: from Functional Genomics to Genome Editing*”, the second volume of this article collection has been published. This reflects the recent progress being made on understanding the ecology, biology, and physiology of plant pathogens as well as on the management of plant diseases.

An increasing world population illustrates the urgency to reduce the losses of agricultural production—caused by biotic and abiotic stress—and to consider food security and food safety as a priority. These are regulated at a global level by the Agenda 2030 “Sustainable Development Goals,” which highlight the need to find solutions to feed world population (zero hunger) and to pay attention to the impact human actions have on the environment. The “Farm to Fork Strategy” is placed at the heart of the “European Green Deal” and fixes two targets for 2030, namely, a 50% reduction in the use of agro-chemicals and to alleviate the serious threat to human health and the environment that these pose.

Crop plants are continuously under attack by pathogens, both before and after harvest, causing economically important crop losses and rendering food security and safety a big challenge to be faced. Knowledge of the pathogen to be controlled and of its epidemiology is essential to find sustainable solutions to defend crops. The availability of new techniques facilitates ever deeper analyses of the genomes of plant pathogens, providing a more reliable resolution in discriminating highly related genetic backgrounds. Kulik et al. used the whole-genome sequencing (WGS) method—whole-genome single-nucleotide polymorphism (SNP) analysis—to identify the pandemic pathogen *Fusarium graminearum sensu stricto*, one of the species responsible for cereal diseases, and to discriminate it from the cryptic species included within the FGSC complex (Xu and Nicholson, 2009), whose diversity is a key factor in defining the dynamics and evolutionary relationships underlying disease outbreaks.

Cereals represent a main source of nutrients for a large part of the human population, and maize global production can be affected by the causal agent of anthracnose *Colletotrichum graminicola*. Becerra et al. re-sequenced the genome of *C. graminicola* M1.001 by using a combination of short-read (Illumina) and long-read (PacBio) technologies, thus revealing new insights into its structure and content. They obtained a chromosome-level assembly, which resulted in a higher density of repetitive elements and RIPs in the dispensable chromosomes (MCs) that could be connected to the adaptation and/or host co-evolution of this fungus.

Crops not conventionally defined as staple foods play an important role for the economy of several countries. A pertinent example is date palm, which is widely cultivated across North Africa, particularly in Tunisia, where 10% of the population is economically dependent on this crop. Rabaaoui et al. used a polyphasic approach to identify *Alternaria* and *Curvularia* strains (pathogens of *Phoenix dactylifera* L.) and to establish their ability to produce mycotoxins, such as alternariol and fumonisin B, with important results achieved in terms of possible risk for consumers.

Oomycetes are fungal-like organisms in the kingdom Chromista that are responsible for several devastating diseases, although some species are studied as biocontrol agents on insects, fungi, and other oomycetes. RNA silencing plays a role in the pathogenicity of *Phytophthora* species. Piombo, Kelbessa et al. made an initial survey of how RNA silencing can affect the regulation of genes involved in pathogenesis and biocontrol. Their work suggests that oomycete sRNAs regulate effectors that may be important in host invasion and that the trans-boundary movement of microRNAs may play a role in biocontrol, thus paving the way for practical applications in the field.

Another important oomycete, which is responsible for significant economic losses due to the lack of efficient disease control, is *Plasmopara viticola*, the causal agent of grapevine mildew, which severely affects viticulture worldwide. Koledenkova et al. reviewed the history, distribution, epidemiology, taxonomy, morphology, reproduction, and infection mechanisms of this pathogen. The authors highlight modern molecular techniques that offer insights into the *P. viticola* susceptibility to control treatments and the genetic aspects of plant resistance, thus giving an update on the strategies available, including sustainable methods such as the use of biocontrol agents (BCAs).

The exploitation of BCAs (Collinge et al., 2022) is receiving more and more interest, and the availability of all the “post-genomics” techniques facilitates the comprehension of the complex and fascinating interactions occurring in the tri-partite plant–pathogen–beneficial organisms system. Piombo, Guaschino et al. analyzed the composition and activity of the secretome of *Clonostachys rosea* to reveal the involvement—as a driven force in fungal adaptation to ecological niches and environmental interactions—of its proteins in mycoparasitic and fungal–plant interactions established by this well-known BCA (Jensen et al., 2022).

*Trichoderma* spp. and *Clonostachys rosea* represent the organisms most frequently used as biocontrol agents (Sarrocco, 2023), with promising results also having been demonstrated in the management of Fusarium head blight on wheat, a disease threatening cereal production and quality due to mycotoxin contamination (Khairullina et al., 2023). However, the application of beneficial organisms can have important ecological consequences due to the release of new isolates in a well-established environment. Alukumbura et al. used a metagenomic approach to study the possible impacts of *Trichoderma gamsii* T6085 on the microbiome associated with wheat, thus demonstrating that T6085 treatment did not cause any community-scale rearrangements. The data revealed the presence of several other taxa to be explored as potential BCAs to integrate with *T. gamsii*.

Advances in genome editing have recently provided new tools to control plant diseases. The CRISPR–Cas9 technique has been used by Lucioli et al. to disrupt the *eIF4E1*gene with the aim of broadening the potato virus Y (PVY) resistance spectrum of *Solanum tuberosum* L. cv. Desirée by pyramiding eIF4E-mediated recessive resistance. The authors explained that the new gene-editing approaches may be profitably used to extend the virus resistance spectrum of elite potato cultivars to preserve their traits.

## Author contributions

All authors listed have made a substantial, direct, and intellectual contribution to the work and approved it for publication.
